# Genome-wide analysis revealed that DZNep reduces tubulointerstitial fibrosis via down-regulation of pro-fibrotic genes

**DOI:** 10.1038/s41598-018-22180-5

**Published:** 2018-02-28

**Authors:** Imari Mimura, Yosuke Hirakawa, Yasuharu Kanki, Ryo Nakaki, Yutaka Suzuki, Tetsuhiro Tanaka, Hiroyuki Aburatani, Masaomi Nangaku

**Affiliations:** 1 Division of Nephrology and Endocrinology, The University of Tokyo Graduate School of Medicine, Bunkyō, Japan; 2 Isotope Science Center, The University of Tokyo, Bunkyō, Japan; 3 Division of Genome Science, Research Center for Advanced Science and Technology, The University of Tokyo, Bunkyō, Japan; 4 Graduate School of Frontier Sciences, The University of Tokyo, Bunkyō, Japan

## Abstract

Tubulointerstitial fibrosis has been recently reported to be caused by the collapse of the epigenetic regulation of kidney diseases. We examined whether pharmacological inhibition of histone modification is effective against renal fibrosis. DZNep (3-deazaneplanocin A) was originally developed as an anti-cancer drug to inhibit the repressive histone mark, H3K27me3. We used a model of chronic tubulointerstitial fibrosis induced by unilateral ischaemia/reperfusion and administered DZNep intravenously to the mice for 8 weeks. We found DZNep contributes to the reduction of tubulointerstitial fibrosis. We selected only tubular cells from *in vivo* samples using laser-capture microdissection because epigenetic regulation is specific to the cell types, and we focused on the changes in the tubular cells. We performed a genome-wide analysis of tubular cells using high-throughput sequencing (RNA-seq) to identify novel epigenetic factors associated with renal fibrosis. We found that pro-fibrotic genes such as *COL3A1* (collagen type 3a1) and *TIMP2* (tissue inhibitor of metalloproteinase 2) were suppressed by DZNep *in vivo*. In addition, pro-fibrotic genes such as *COL4A1* (collagen type 4a1), *TIMP2* and *MMP14* were down-regulated by DZNep *in vitro*. In conclusion, we found that pharmacological epigenetic modification by DZNep decreased the expression levels of fibrogenic genes in tubular cells and inhibited tubulointerstitial fibrosis.

## Introduction

After acute kidney injury (AKI), kidneys have generally been believed to regain complete renal function, with a positive long-term prognosis for renal function after surviving the acute crisis. However, recent data from epidemiological studies have revealed that AKI may develop into subsequent chronic kidney diseases (CKDs), leading to end-stage renal disease (ESRD). AKI-to-CKD transition has been demonstrated to occur through maladaptive repair after recovery from AKI, with the underlying molecular mechanisms^1^ shown below. There are several mechanisms of AKI-to-CKD transition, including tubulointerstitial hypoxia. Hypoxia in the tubulointerstitium has been reported to induce fibrosis over the course of years^2–10^. Hypoxia-inducible factor-1 (HIF-1) is a master regulator of its downstream genes under hypoxic conditions^11–13^. Tubulointerstitial hypoxia deteriorates fibrosis in the kidney. In this study, we examined the effect of DZNep (3-deazaneplanocin A) on the tubular fibrosis that mediates AKI-to-CKD transition. To examine whether tubular fibrosis is affected by DZNep through molecular mechanisms such as increased eNOS or alteration of the expression of downstream genes under hypoxia, we administered DZNep to animal models for kidney diseases, and we identified sets of genes that play important roles in tubular fibrosis via a genome-wide analysis of RNA-seq.

## Results

### Ischaemia/reperfusion injury led to tubulointerstitial fibrosis, and DZNep reduces tubulointerstitial fibrosis in ischaemia/reperfusion injury

**Figure 1:**
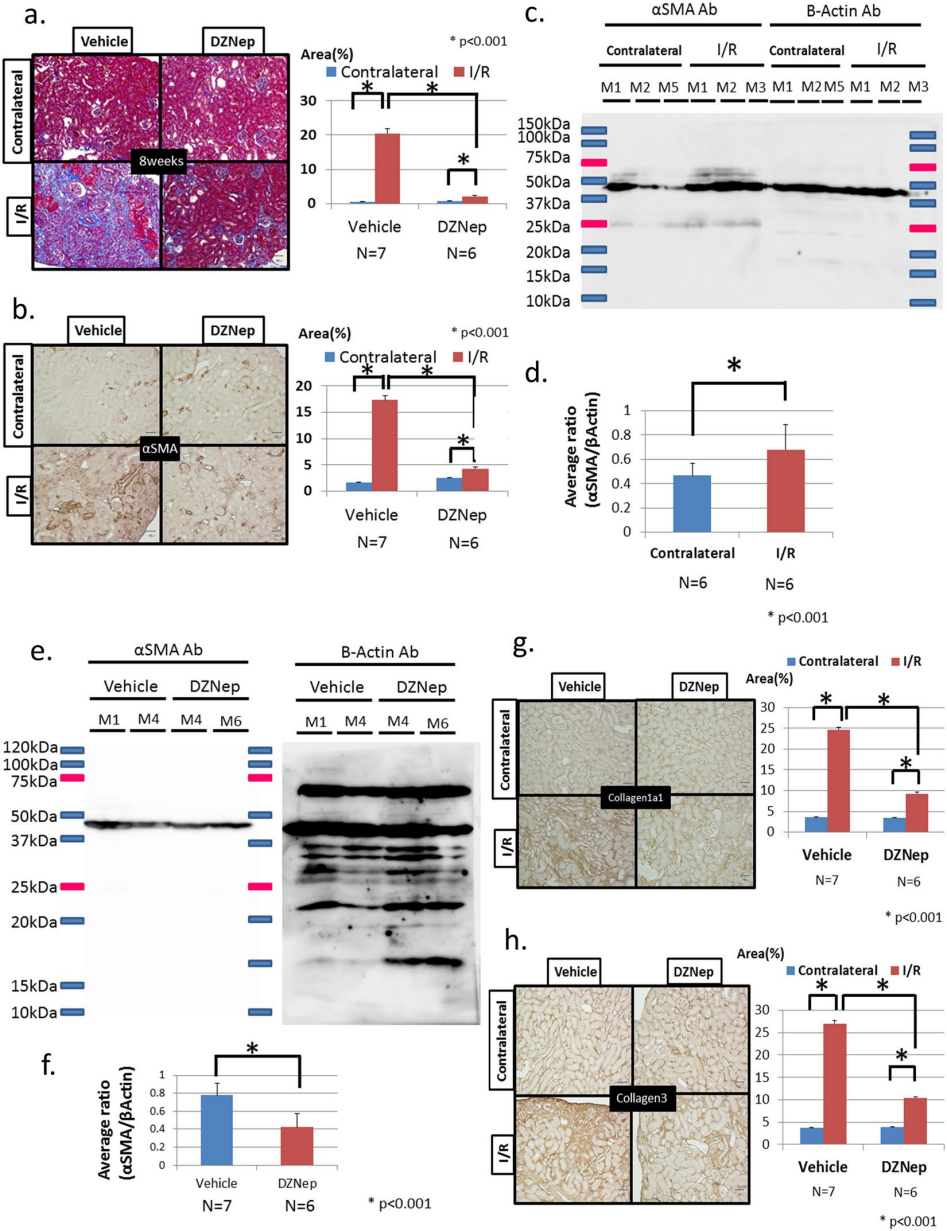
(**a**) Masson trichrome staining (left). The right kidney was the contralateral and the left kidney had unilateral I/R injury. I/R model mice were administered the vehicle or DZNep for 8 weeks. The quantitative analysis of the fibrotic area (%) is shown on the right. The vehicle group had seven mice (n = 7), and the DZNep group had six mice (n = 6). (**b**) Immunohistochemistry of α-smooth muscle actin staining (left). I/R model mice were administered the vehicle or DZNep for 8 weeks. The quantitative analysis of the fibrotic area (%) is shown on the right. (**c**) Representative results of the whole membrane blotted mouse renal cortex from each group (contralateral kidney: M1, M2, M5; I/R injury kidney: M1-M3). (**d**) Quantitative analysis using densitometry for the western blotting of α-smooth muscle actin (αSMA) corrected by β-actin as an internal control. We show the average α-SMA/βactin ratio in the contralateral kidneys (N = 6) and the I/R injury kidneys (N = 6). (**e**) Representative results of the whole membrane blotted mouse renal cortex from each group (vehicle group; M1, M4; DZNep group: M4, M6). (**f**) Quantitative analysis for the western blotting of αSMA corrected by β-actin using densitometry is shown. We showed the average αSMA/β-actin ratios in the vehicle group (N = 7) and the DZNep group (N = 6). (**g**) Immunohistochemistry of collagen type 1 alpha 1 (Col1A1) (left). I/R model mice were administered with vehicle or DZNep for 8 weeks. Quantitative analysis of fibrotic area (%) was shown in the right side. (**h**) Immunohistochemistry of collagen type 3 (left). I/R model mice were administered the vehicle or DZNep for 8 weeks. Quantitative analysis of fibrotic area (%) is shown on the right.

We generated ischaemia/reperfusion (I/R) injury model mice and observed tubulointerstitial fibrosis after 8 weeks. Although mild I/R injury has been well known as an acute and reversible kidney injury model, we have found that the development of tubulointerstitial fibrosis after 8 weeks of severe I/R injury can serve as a model of AKI to CKD transition. To clarify the effects of epigenetic modulation on AKI to CKD transition after I/R injury, we administered DZNep intravenously once a week after surgery for 8 weeks (details in materials and methods). The serum blood urea nitrogen concentration in each group was 33.2 ± 2.04 mg/dl (vehicle group) and 30.3 ± 2.03 mg/dl (DZNep group), and the serum creatinine concentrations of each group were 0.33 ± 0.09 mg/dl (vehicle group) and 0.37 ± 0.09 mg/dl (DZNep group). There were no significant differences between the two groups. However, quantitative analysis of Masson trichrome staining showed an 89.8% reduction of fibrotic areas in the DZNep group compared with the vehicle group with I/R injury (Fig. 1a). Immunohistochemical staining of α-smooth muscle actin (αSMA) as a marker of tubulointerstitial fibrosis also showed similar results in the DZNep group with I/R injury (Fig. 1b). αSMA was reduced by 75.3% in the DZNep group compared with the vehicle group with I/R injury. We examined the αSMA protein level in the kidney cortex in both contralateral kidneys and I/R injury kidneys. We show a representative western blot in Fig. 1c. The quantitative western blot analysis is shown in Fig. 1d (contralateral kidneys, N = 6; I/R kidneys, N = 6). The average ratio of αSMA corrected by β-actin showed a 45.1% increase in I/R injury kidneys compared with contralateral kidneys. Next, we found that the αSMA protein level decreased in the DZNep group, as shown in Fig. 1e. The quantitative western blot analysis in both groups showed a 45.8% reduction in the DZNep group (n = 6) compared with vehicle group (n = 7) (Fig. 1f).

**Figure 2:**
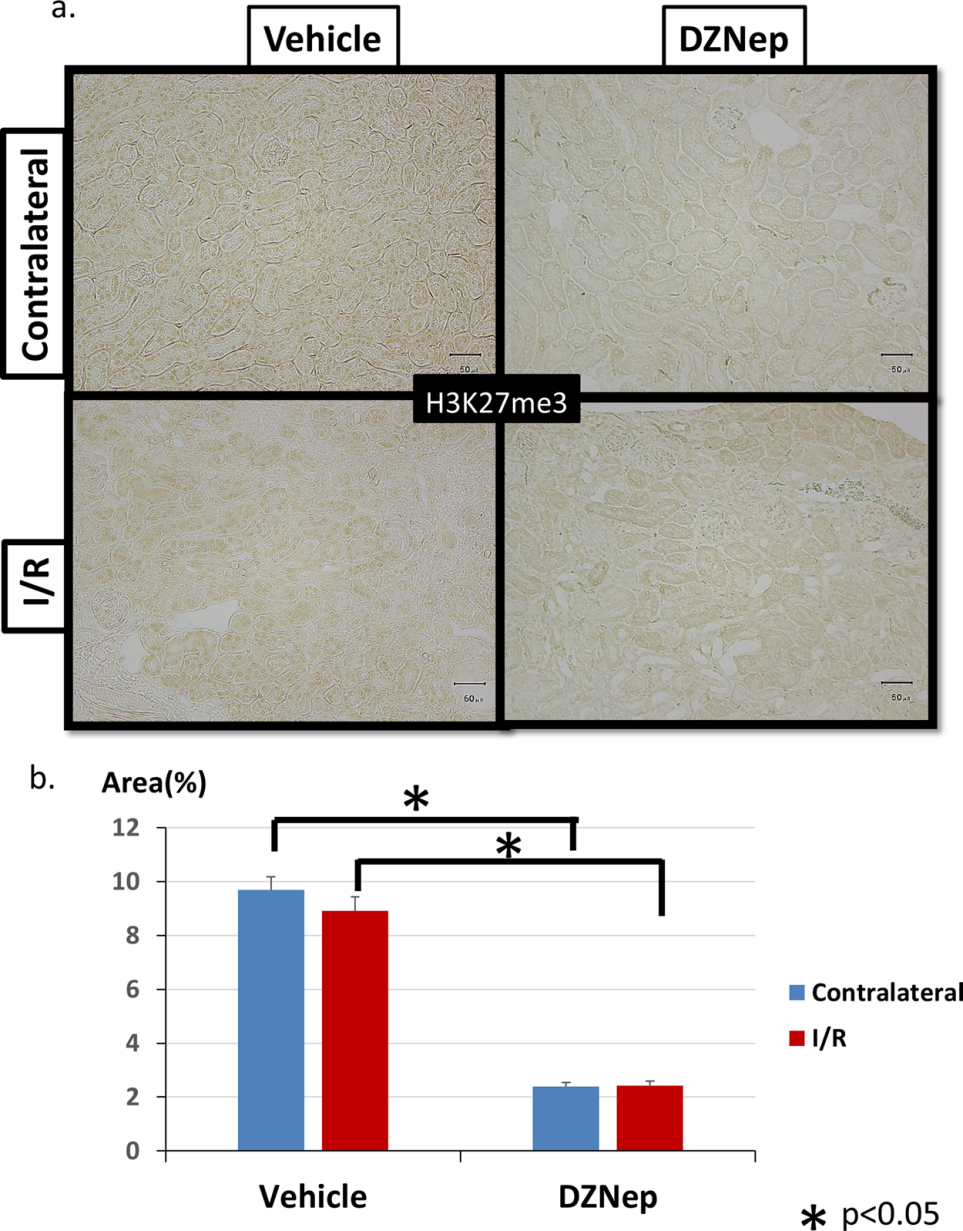
(**a**) Immunohistochemistry of H3K27me3 staining. I/R model mice were administered the vehicle or DZNep for 8 weeks. (**b**) Quantitative analysis of H3K27me3-positive tubular cells in I/R model mice administered the vehicle or DZNep for 8 weeks.

The immunohistochemical staining of collagen type 1 and collagen type 3 showed similar reductions in the DZNep group. Collagen type 1 was reduced by 62.4% (Fig. 1g), and collagen type 3 was reduced by 61.6% (Fig. 1h) compared with the vehicle group. These results consistently demonstrated that DZNep reduces tubulointerstitial fibrosis in I/R injury model mice. To clarify which cells in the kidney are the targets of DZNep, we used immunohistochemistry with the H3K27me3 antibody. As shown in Fig. 2a, the nuclear staining of H3K27me3 in tubular cells was eliminated by administration of DZNep, demonstrating that DZNep reduces the histone modification by H3K27me3 in the nucleus of tubular cells. The expression level of H3K27me3 in I/R injury kidneys did not change compared with contralateral kidneys because it was not influenced by I/R injury. This may be because the expression level of Ezh2 *in vivo* did not change between contralateral kidneys and I/R kidneys. Quantitative analysis for H3K27me3-positive tubular cells revealed a significant reduction of positive cells in the DZNep group compared with the vehicle group in both contralateral kidneys and I/R kidneys (Fig. 2b).

### Genome-wide analysis of tubular cells in I/R models with DZNep

**Figure 3:**
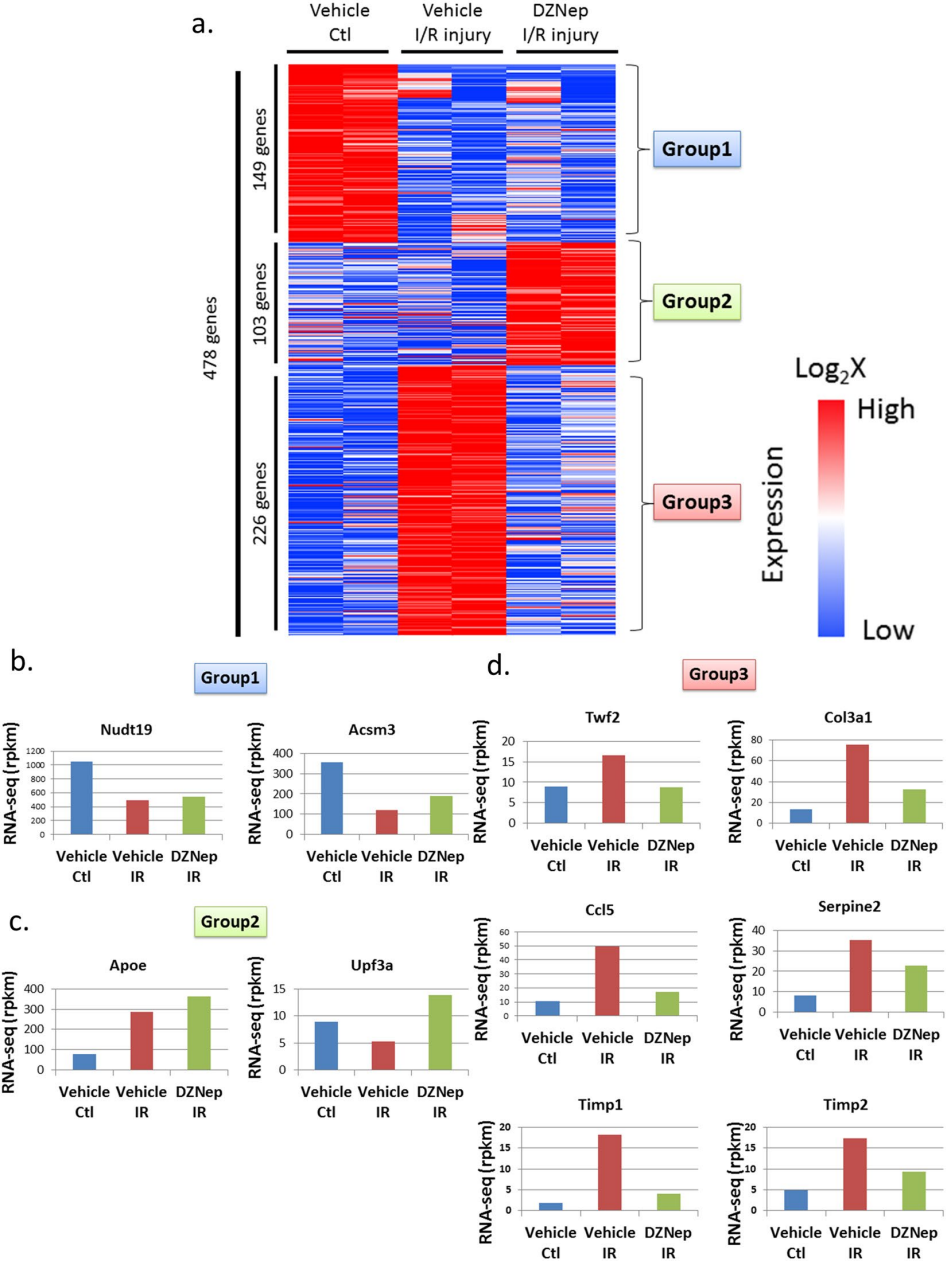
(**a**) Heatmap summarizing the RNA-seq data. In total, 478 genes were selected and classified into 3 groups. We used two I/R injury model mice in each group (N = 2) and extracted right kidneys (contralateral), left kidneys (I/R injury) with vehicle administration and left kidneys (I/R injury) with DZNep administration. (**b**) The examples of genes that belong to group 1. (**c**) The examples of genes that belong to group 2. (**d**) The examples of genes that belong to group 3.

**Figure 4:**
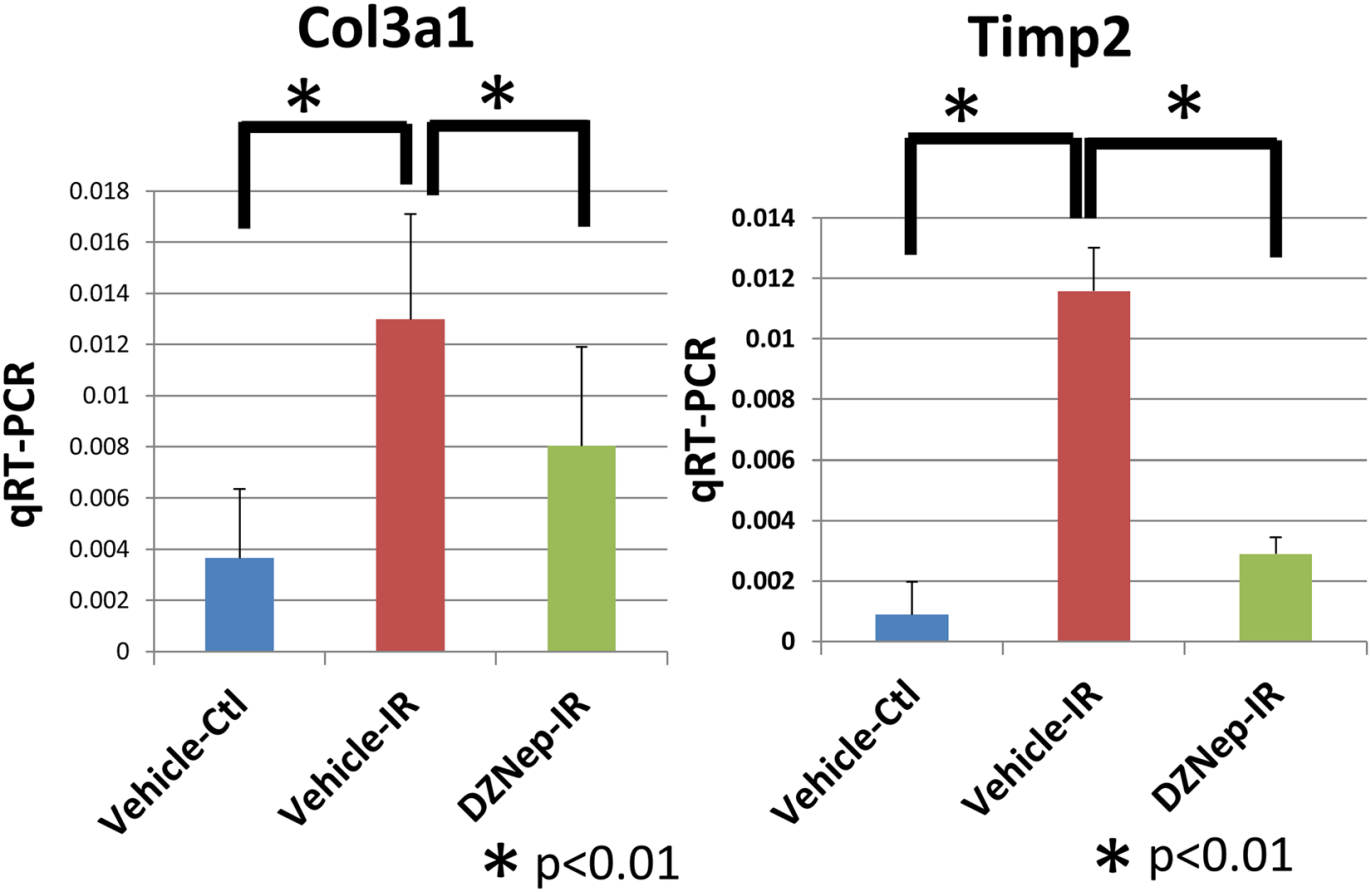
Validation of RNA-seq using qRT-PCR for Col3a1 and Timp2 using *in vivo* samples. We extracted mRNA of renal cortex from

**Figure 5:**
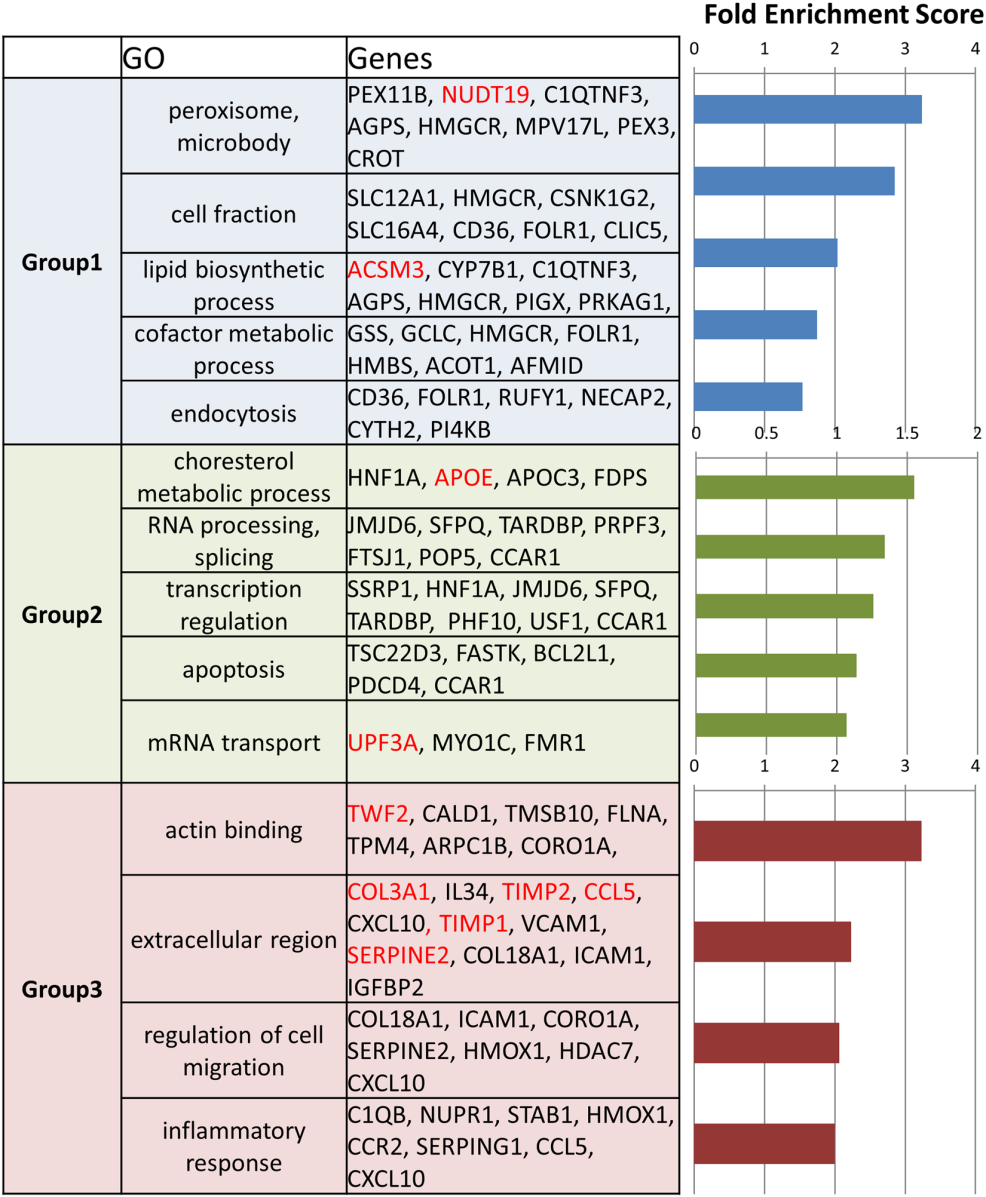
Functional annotations for the 3 groups. Representative gene symbols for each category are shown in the middle panel. Fold enrichment scores for each category from DAVID are shown in the bar graphs in the right panel.

As the kidney is composed of various cell types, it is important to specifically analyse the target cells of the study. We performed RNA-seq on tubular cells that were extracted from the experimental mouse kidney by laser-capture microdissection (details are shown in material and methods). To examine gene expression patterns of the I/R model treated with DZNep, we constructed a heat map, shown in Fig. 3a. We prepared 2 *in vivo* samples for each treatment (vehicle contralateral, vehicle I/R injury and DZNep I/R injury). To identify the genes regulated by DZNep, we set the criteria for the listed genes at >1.5 log fold higher than the other 2 conditions and the maximum FPKM (fragments per kilobase of exon per million fragments mapped) was more than 20 in each condition. Four hundred and seventy-eight genes were listed in the heat map. According to the expression patterns, we classified them into 3 groups. We listed all the genes for group 1–3 in Supplementary Table 1. Group 1 included 149 genes that were down-regulated in vehicle I/R injury and DZNep I/R injury treatments compared to the vehicle contralateral treatment. Group 2 included 103 genes that were up-regulated in the DZNep I/R injury treatment compared to in the other 2 conditions. Group 3 included 226 genes that were up-regulated in the vehicle I/R injury treatment compared with the vehicle contralateral treatment and down-regulated in the DZNep I/R injury treatment compared to the vehicle I/R injury treatment. The representative gene expression patterns of each group are shown in Fig. 3b–d. For example, the expression levels of *Nudt19* (nucleoside diphosphate-linked moiety X)-type motif 19 and *Acsm3* (acyl-CoA synthetase medium-chain family member 3) were down-regulated in the I/R injury model both with and without DZNep (Fig. 3b). The expression levels of *Apoe* (apolipoprotein E) and *Upf3a* (UPF3 regulator of nonsense transcripts homologue A) were up-regulated in I/R injury model mice with DZNep compared with those without DZNep (Fig. 3c). The expression patterns of *Twf2* (twinfilin actin-binding protein 2), *Col3a1* (collagen type 3a1), *Ccl5* (chemokine ligand 5), *Timp1* (tissue inhibitor of metalloproteinase 1) and *Timp2* (tissue inhibitor of metalloproteinase 2) are shown in Fig. 3d. The expression levels of group 3 in I/R injury model mice were up-regulated compared to in the vehicle mice, and down-regulated in the I/R injury with DZNep mice compared with those without DZNep. We validated the RNA-seq results of *Col3a1* and *Timp2* by qRT-PCR. The expression levels of those genes in the I/R injury model mice were significantly up-regulated compared to the vehicle mice, and the expression levels in I/R injury model mice with DZNep were also significantly down-regulated compared to those without DZNep (Fig. 4). Next, we subjected those genes to ontology analysis with the Database for Annotation, Visualization and Integrated Discovery (DAVID) to investigate the related biological processes. As shown in Fig. 5, the genes associated with actin binding including *TWF2* (twinfilin actin-binding protein 2), *CALD1* (caldesmon 1) and *CORO1A* (coronin actin-binding protein 1A) were most enriched in group 3. Pro-fibrotic genes including *Col3a1*, *Timp1*, and *Timp2* were the second most enriched genes in group 3. Genes associated with the inflammatory response were also significantly enriched in group 3.

### Genome-wide analysis of hypoxia-inducible and DZNep-responsive genes in cultured tubular cells

**Figure 6:**
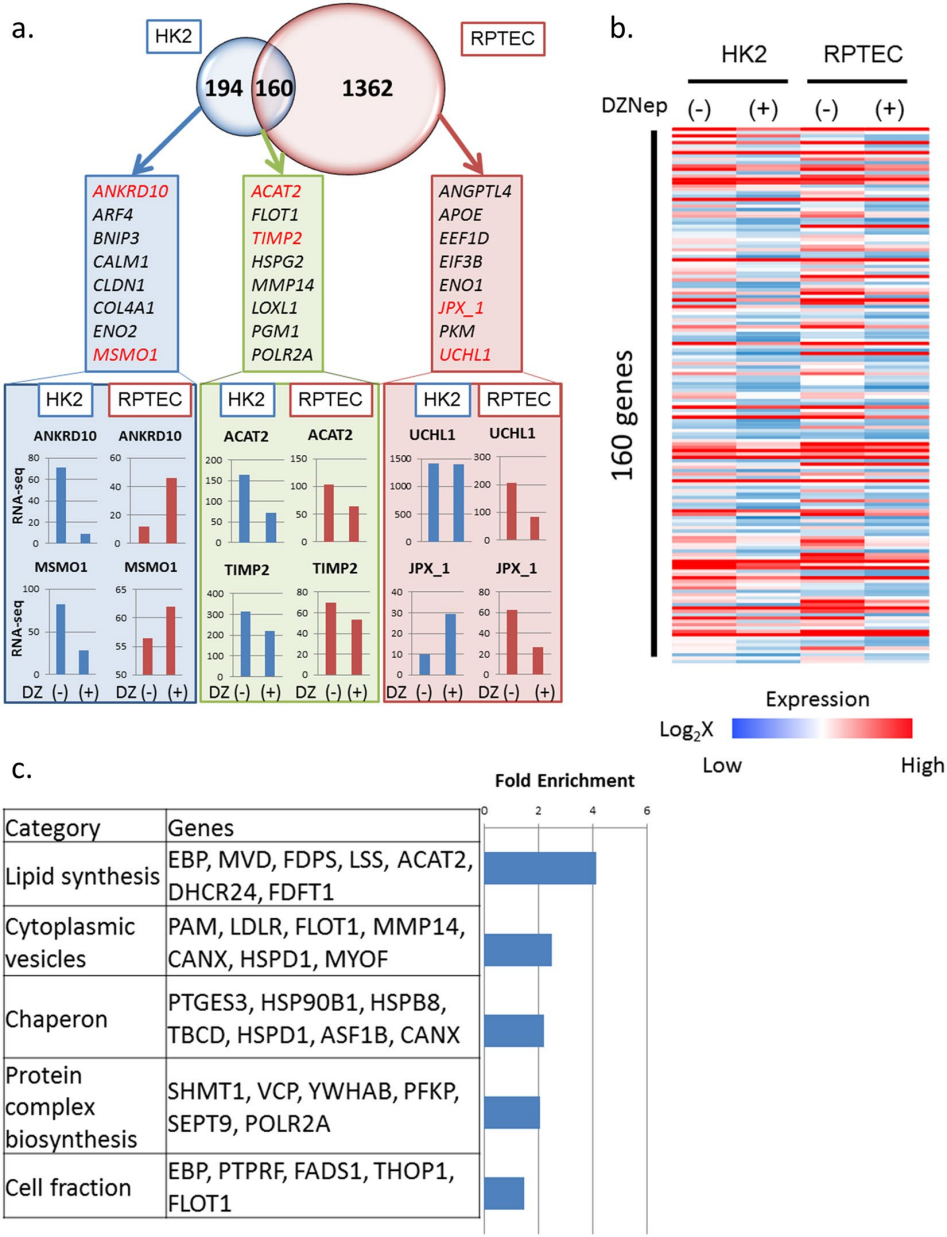
(**a**) Venn diagram of DZNep target candidates in HK2 and RPTEC from RNA-seq data. The numbers indicate the unique HK2, unique RPTEC and common DZNep target candidates. (**b**) Heatmap of 160 genes whose expression levels are decreased by DZNep administration in both HK2 and RPTEC. (**c**) Functional annotations for commonly down-regulated genes by DZNep. The representative gene symbols for each category are shown in the middle panel. The enrichment scores of each category from DAVID are shown in the bar graphs in the right panels.

To identify important targets by DZNep, we performed an *in vitro* experiment using cultured renal tubular cells, human kidney-2 (HK2) cells, and renal proximal tubular epithelial cells (RPTEC). We exposed these cells to DZNep for 48–72 hours at the concentration of 1 μM and performed RNA-seq to find the target genes of DZNep. As shown in Fig. 6a, we compared the DZNep target genes in both cells. We set the criteria for the downstream DZNep targets at >1.5 log fold higher with DZNep than without DZNep. In addition, the RPKM value of the hypoxic condition was >20.0. Three hundred and fifty-four genes met the criteria in HK2 cells and 1,522 genes in RPTEC cells. We show all those gene names in Supplementary Table 2. We found 194 genes that were up-regulated by hypoxia only in HK2 cells and 1362 genes that were up-regulated by hypoxia only in RPTEC cells. One hundred sixty genes, including *ACAT2* (acetyl-CoA acetyltransferase 2) and *TIMP2*, were commonly up-regulated by DZNep in both types of cultured tubular cells. The representative genes that were down-regulated by DZNep only in HK2 cells included *ANKRD10* (ankyrin repeat domain 10) and *MSMO1* (methylsterol monooxygenase) and are shown in the left blue panel. The representative genes that were down-regulated by DZNep only in RPTEC cells included *UCHL1* (ubiquitin C-terminal hydrolase L1) and *JPX_1* (Jpx transcript, Xist activator) and are shown in the right red panel. The representative genes that were down-regulated by DZNep both in HK2 and RPTEC cells included *ACAT2* and *TIMP2* and are shown in the middle green panel (Fig. 6a).

**Figure 7:**
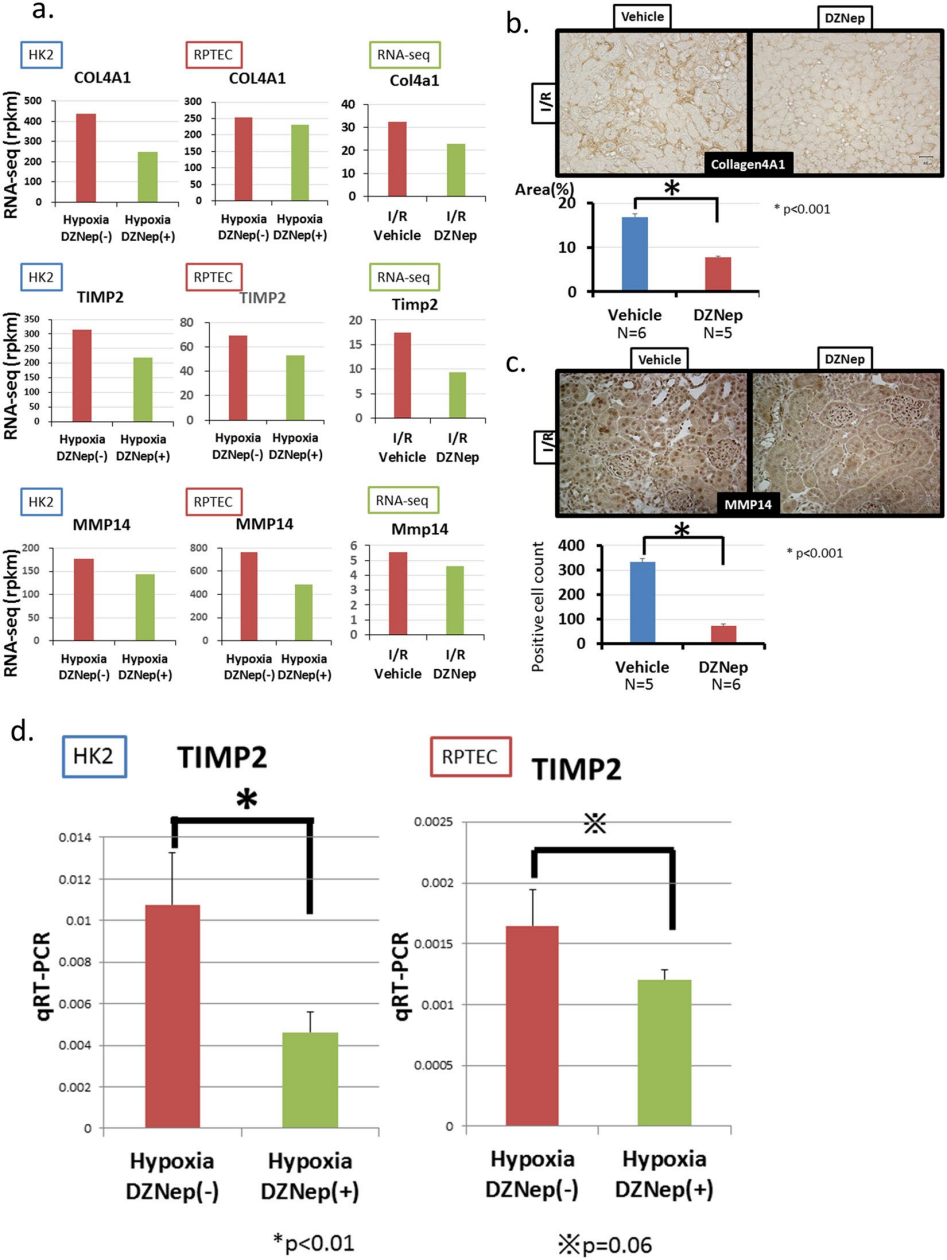
(**a**) The results of RNA-seq of fibrosis-associated genes including COL4A1, TIMP2 and MMP14 in HK2 (left column), RPTEC (middle column) and *in vivo* samples (right column). (**b**) Immunohistochemistry of collagen type 4 staining for I/R injured mice of both groups. The quantitative analysis is shown for each group (vehicle; N = 6, DZNep; N = 5). (**c**) Immunohistochemistry of MMP14 staining for I/R injured mice of both groups. The quantitative analysis of MMP14-positive cell counts is shown for each group (vehicle; N = 5, DZNep; N = 6). (**d**) Validation of RNA-seq using qRT-PCR for TIMP2.

We focused on 160 genes that were down-regulated by DZNep in both HK2 and RPTEC cells because they were the target candidates of DZNep in two types of cells. Their expression patterns are shown in Fig. 6b. The functional annotations of these DZNep target candidates are shown in Fig. 6c. Lipid synthesis-associated genes including *EBP* (emopamil-binding protein) and *ACAT2* were the most enriched out of these genes. Cytoplasmic vesicles-associated genes including *PAM* (peptidylglycine alpha-amidating monooxygenase), *LDLR* (low density lipoprotein receptor) and *MMP14* were also highly enriched. The representative genes that were down-regulated by DZNep in both cells are shown in Fig. 7a (left column and middle column). Pro-fibrotic genes including *COL4A1, TIMP2* and *MMP14* were down-regulated by DZNep. We also confirmed that these genes were down-regulated in the DZNep group with I/R models *in vivo* (Fig. 7a, right column). In addition, we performed immunohistochemistry of *Col4a1* and *Mmp14 in vivo* (Fig. 7b,c). The protein level of *Col4a1* significantly decreased in the DZNep group of the I/R model (Fig. 7b). The positive cell counts of *Mmp14* in the DZNep group of the I/R models significantly decreased compared with the vehicle group (Fig. 7c).

To validate the results of RNA-seq, we performed qRT-PCR of *TIMP2* under hypoxic condition with or without DZNep (Fig. 7d). In HK2 cells, the expression of *TIMP2* was significantly down-regulated by DZNep. In RPTEC cells, the expression of *TIMP2* was also reduced by DZNep (p value = 0.06). We confirmed that DZNep decreased the expression of pro-fibrotic genes in renal tubular cells, leading to the amelioration of tubulointerstitial fibrosis.

**Figure 8:**
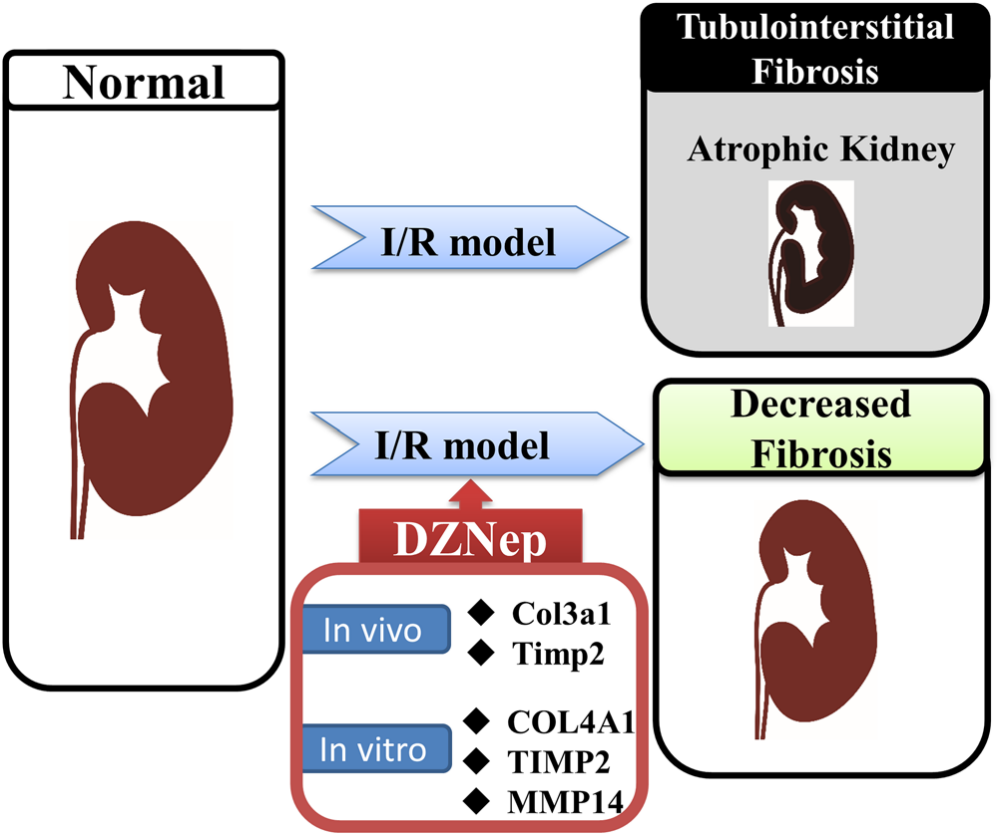
Schematic diagram of renal fibrosis ameliorated by DZNep.

We show a summarizing schematic diagram in Fig. 8. I/R injury leads to tubulointerstitial fibrosis, resulting in atrophic kidneys. However, administration of DZNep contributes to the reduction of tubulointerstitial fibrosis. DZNep inhibits the expression of pro-fibrotic genes such as *Col3a1, Timp2 in vivo* and *COL4A1*, *TIMP2* and *MMP14 in vitro*.

## Discussion

We found that DZNep inhibits tubulointerstitial fibrosis using a murine model of AKI-to-CKD transition. DZNep is a histone methyltransferase inhibitor and disrupts polycomb-repressive complex 2 (PRC2)^14^. PRC2 contains 3 core proteins, EZH2 (enhancer of zeste homologue 2 polycomb repressive complex 2 subunit), SUZ12 (SUZ12 polycomb repressive complex 2 subunit) and EED (embryonic ectoderm development). The SET (suppressor of variegation-enhancer of zeste-trithorax) domain of EZH2 mediates the histone methyltransferase activity. DZNep was originally developed as an anti-tumour drug^15^. DZNep has been reported to induce apoptosis in tumours including acute myeloid leukaemia^16^. DZNep treatment significantly induces erythroid differentiation of K562 cells^17^. DZNep plays a role not only in blood cells, but DZNep also inhibits the proliferation of colon cancer HCT116 cells by inducing cellular senescence and apoptosis^18^.

Another report has shown that *Ezh2* expression is increased in cultured endothelial cells (ECs) exposed to hypoxia and in ECs extracted from ischaemic mouse limb muscles^19^. DZNep increased eNOS and BDNF mRNA and protein levels and enhanced migration and angiogenesis of ECs under normoxia and hypoxia. DZNep increased circulating levels of pro-angiogenic haematopoietic cells and blood flow recovery.

There have been some papers investigating the relationship between DZNep and fibrosis. For example, Ezh2 has been recently reported to enhance the differentiation of fibroblasts into myofibroblasts^20^. The authors demonstrated that the inhibition of Ezh2 by DZNep reduced the TGFβ1-induced differentiation of human lung fibroblasts into myofibroblasts and that DZNep inhibited Smad2/3 nuclear translocation without affecting Smad2/3 phosphorylation. On the other hand, DZNep has been reported to induce dermal fibrosis by repressing the expression of fra-2^21^. Inhibition of H3K277me3 stimulated the release of collagen in cultured fibroblasts, and treatment with DZNep exacerbated fibrosis by overexpression of constitutively active TGFβ1. They also demonstrated that knockdown of fra-2 prevented the pro-fibrotic effects of DZNep. These results suggest that DZNep might act as a pro-fibrotic factor in other organs including the dermis.

However, in the kidney, Zhou X *et al*. found increased expression of *Ezh2* and H3K27me3 in renal cultured fibroblasts and in fibrotic kidneys from unilateral urethral obstruction (UUO) model mice^22^. Not only DZNep but siRNA-mediated silencing of Ezh2 inhibited TGFβ1-induced activation of renal interstitial fibroblasts. They clarified that DZNep inhibited renal fibroblast activation by evaluating markers including α-SMA, collagen I, and fibronectin in NRK49F cells. Moreover, DZNep inhibited the activation of the TGFβ1 signalling pathway in the UUO model mice. In addition, they demonstrated that DZNep inhibits UUO injury-induced EGFR and PDGFRβ phosphorylation and that DZNep increases phosphatase and tensin homologue (PTEN) expression, along with reducing the number of renal tubular cells arrested at the G2/M phase of the cell cycle. The improvement of fibrosis in the UUO model by DZNep as described above is consistent with our findings observed in the fibrosis model after ischaemia-reperfusion.

We identified the down-regulation of pro-fibrotic genes such as *MMP14* by DZNep using cultured tubular cells. The responses to hypoxia of two cell lines, HK2 and RPTEC, were different, as shown in Fig. 6a. This may be because HK2 is an immortalized cell line, while RPTEC is a primary cultured cell line. However, 160 genes including *MMP14* were commonly down-regulated by DZNep in both cell lines.

MMP family members have a common domain structure, and the crystal structure of MMP14 has been clarified^23^. MMP2 and MMP14 are mainly produced in fibroblasts. MMP-14 is also known as MT1-MMP (membrane type 1-matrix metalloproteinase)^24^. It is a zinc-transmembrane metalloprotease involved in the degradation of the extracellular matrix and tumour invasion^25^. MMP14 has been reported to interact with TIMP2^26–29^. TIMP2 binds to the zinc catalytic site of MMP14^28^. In addition, the C-terminal domain of TIMP2 participates in binding to MMP14. In this study, both TIMP2 and MMP14 were down-regulated by DZNep in HK2 and RPTEC cells. Our results suggest that these are the targets of DZNep to reduce tubulointerstitial fibrosis in AKI-to-CKD transition.

DZNep reduces EZH2 levels and inhibits the trimethylation of lysine 27 on histone H3 (H3K27me3)^30^. DZNep has been reported to up-regulate target genes by inhibiting the repressive histone mark, H3K27me3. In this study, we clarified that pro-fibrotic genes were down-regulated by DZNep in both *in vivo* and *in vitro*. Whether the pro-fibrotic genes up-regulated by DZNep are suppressed by histone modification or other mechanisms such as regulation by microRNAs (miRs)^31,32^, is a subject for future studies.

In summary, we demonstrated that epigenetic modulation by DZNep improved tubulointerstitial fibrosis in a mouse model of AKI-to-CKD transition. Our results suggest a crucial role of epigenetic changes in AKI-to-CKD transition, which can be an appropriate target of therapeutic approaches.

## Materials and Methods

### Animal Experiment

All animal experimental protocols in this study were approved by the Ethical Committee on Animal Experiments of the University of Tokyo (P13–016), and all animal experiments were conducted in accordance with the institutional guidelines of the University of Tokyo.

To investigate the level of renal fibrosis in a fibrotic kidney model, 11-week-old C57BL/6 J male mice (Nippon Bio-Supp. Center, Saitama, JAPAN) weighing 20–25 g were used. All the animals were housed in cages in a temperature- and light-controlled environment in an accredited animal care facility, with free access to normal chow and tap water. After 3–7 days of acclimatization, mice underwent ischaemia/reperfusion surgery. For surgery, general anaesthesia was accomplished with a 40 mg/kg intraperitoneal injection of pentobarbital, and 10 mg/kg pentobarbital was added if necessary. Under general anaesthesia, a median incision was made, and the abdomen was opened. The left renal artery was exposed, and ischaemia was induced by clip for 30 minutes. The colour of the kidney was carefully monitored to determine whether ischaemia was sufficiently induced, and the clip was released. In this experiment, the mice of the vehicle group were anaesthetized for the same amount of time. We administered DZNep to these mice 1 hour after surgery. DZNep was dissolved in DMSO and administered intravenously at the dose of 0.07 mg/kg. Vehicle animals received DMSO with phosphate-buffered saline (PBS). DZNep was administered on days 0, 2, 4, and the consecutively once a week for 8 weeks after surgery. After 8 weeks, the kidneys were harvested, and tissue mRNA was subjected to laser-capture microdissection to collect only tubular cells.

### Laser-Capture Microdissection

Kidney samples were embedded in OCT compound (Sakura Fine Technical, Tokyo, Japan), frozen in liquid nitrogen, and stored at −80 °C until use. The sections (4 μm) were fixed with Giemsa stain for 30 seconds. After washing them twice with 100% ethanol for one minute, the sections were fixed with xylene for 3 minutes. We cut the tubular cells using the PALM MicroBeam (Zeiss, Germany) system.

### Cell Culture

HK2 (human kidney-2: ATCC CRL-2190) cells were purchased from ACTT (Summit Pharmaceuticals International Corporation, Tokyo, Japan). They were cultured in Dulbecco’s modified Eagle’s medium with F12 (Wako, Osaka, Japan) supplemented with 10% heat-inactivated foetal bovine serum (FBS). Renal proximal tubular epithelial cells (RPTECs: CC2553) (Lonza, Tokyo, Japan) were cultured in EVM supplemented with 0.5% FBS. Cells were grown in a humidified atmosphere with 5% CO_2_ at 37 °C. The hypoxic condition (1% O_2_ for 24 hours) was brought about by means of a hypoxic cultivation incubator (APM-30D, ASTEC, Fukuoka, Japan).

### Immunohistochemical Analyses

Kidney samples were harvested and fixed with 10% formalin overnight. Paraffin-embedded sections (3 μm) were stained by the indirect immunoperoxidase method. We use anti-collagen I antibody (1:400; Abcam), rabbit anti-collagen III antibody (1:500; Abcam), mouse anti-α-smooth muscle actin monoclonal antibody ASM-1 (1:500, Progen Biotechnik. Heidelberg, Germany), and anti-H3K27me3 (Wako, Osaka, Japan) as the primary antibodies.

### Western Blotting

Renal cortex tissue was washed with ice-cold PBS, homogenized with a homogenizer, and lysed with RIPA buffer, as described previously. The membrane was blocked with 1% skim milk with TBS-T and incubated with a primary antibody against α-smooth muscle actin (Cell Signaling Technology, MA, USA), and β-actin (Cell Signaling Technology, MA, USA). The secondary antibody was anti-rabbit IgG (170–6515, Bio-Rad, USA).

### RNA Isolation and Reverse Transcription PCR

Total cellular RNA was isolated using RNAiso Plus (Takara, Shiga, Japan) according to the manufacture’s protocol. First-strand cDNA was synthesized utilizing the PrimeScript RT reagent kit (Perfect Real Time) (Takara, Shiga, Japan).

### RT-qPCR

The purified mRNA or ChIP samples were quantified by qPCR. qPCR was performed by the KAPA SYBR FAST qPCR Kit (Nippon Genetics, Tokyo, Japan) on the CFX96 Touch (Bio-Rad Hercules, CA). The expression level of each gene was normalized by β-actin (ACTB). The sequences of the primers used for qPCR are listed in Supplementary Table 3.

### RNA-seq sample analysis

We isolated mRNA as described above. RNA-seq libraries were prepared and sequenced using the HiSeq platform (Illumina, San Diego, CA) according to the manufacturer’s protocol. The reads per kilobase of exons per million mapped reads (RPKM) of each gene were calculated based on the length of the gene and the read counts mapped to the gene. The sequences were aligned using the human reference genome (UCSC hg19) with ELAND (Illumina, San Diego, CA)^31^. The details were described in our previous paper^32,33^.

### Data access

Data were analysed according to the minimum information about a microarray experiment (MIAME) guidelines. The annotations of the probe numbers and targeted sequences are shown on the Affymetrix web page. The data used in this publication are accessible through the National Center for Biotechnology Information and the Gene Expression Omnibus (DRA003786 and DRA003787) for RNA-seq.

### Statistical analysis

Data shown are the mean ± S.E., and *p* values were calculated using two-tailed unpaired Student’s t test. p < 0.05 was considered significant.

## Electronic supplementary material

Supplementary Information

## References

[1] Basile DP (2016). Progression after AKI: Understanding Maladaptive Repair Processes to Predict and Identify Therapeutic Treatments. J Am Soc Nephrol.

[2] Mimura I (2010). Cytoglobin, a novel globin, plays an antifibrotic role in the kidney. Am J Physiol Renal Physiol.

[3] Tanaka T (2006). Hypoxia and expression of hypoxia-inducible factor in the aging kidney. J Gerontol A Biol Sci Med Sci.

[4] Tanaka T (2005). Hypoxia-inducible factor modulates tubular cell survival in cisplatin nephrotoxicity. Am J Physiol Renal Physiol.

[5] Tanaka T (2005). Induction of protective genes by cobalt ameliorates tubulointerstitial injury in the progressive Thy1 nephritis. Kidney Int.

[6] Manotham K (2004). Transdifferentiation of cultured tubular cells induced by hypoxia. Kidney Int.

[7] Tanaka T, Miyata T, Inagi R, Fujita T, Nangaku M (2004). Hypoxia in renal disease with proteinuria and/or glomerular hypertension. Am J Pathol.

[8] Tanaka T (2005). Cobalt promotes angiogenesis via hypoxia-inducible factor and protects tubulointerstitium in the remnant kidney model. Lab Invest.

[9] Nangaku M (2006). Chronic hypoxia and tubulointerstitial injury: a final common pathway to end-stage renal failure. J Am Soc Nephrol.

[10] Mimura I, Nangaku M (2010). The suffocating kidney: tubulointerstitial hypoxia in end-stage renal disease. Nat Rev Nephrol.

[11] Wang GL, Semenza GL (1995). Purification and characterization of hypoxia-inducible factor 1. J Biol Chem.

[12] Semenza GL, Koury ST, Nejfelt MK, Gearhart JD, Antonarakis SE (1991). Cell-type-specific and hypoxia-inducible expression of the human erythropoietin gene in transgenic mice. Proc Natl Acad Sci USA.

[13] Semenza GL (2010). HIF-1: upstream and downstream of cancer metabolism. Curr Opin Genet Dev.

[14] Fiskus W (2009). Combined epigenetic therapy with the histone methyltransferase EZH2 inhibitor 3-deazaneplanocin A and the histone deacetylase inhibitor panobinostat against human AML cells. Blood.

[15] Mayr C (2015). 3-Deazaneplanocin A May Directly Target Putative Cancer Stem Cells in Biliary Tract Cancer. Anticancer Res.

[16] Zhou J (2011). The histone methyltransferase inhibitor, DZNep, up-regulates TXNIP, increases ROS production, and targets leukemia cells in AML. Blood.

[17] Fujiwara T (2014). 3-Deazaneplanocin A (DZNep), an inhibitor of S-adenosylmethionine-dependent methyltransferase, promotes erythroid differentiation. J Biol Chem.

[18] Sha M (2015). DZNep inhibits the proliferation of colon cancer HCT116 cells by inducing senescence and apoptosis. Acta Pharm Sin B.

[19] Mitic T (2015). EZH2 modulates angiogenesis *in vitro* and in a mouse model of limb ischemia. Mol Ther.

[20] Xiao, X. *et al*. EZH2 enhances the differentiation of fibroblasts into myofibroblasts in idiopathic pulmonary fibrosis. *Physiol Rep* **4**, 10.14814/phy2.12915 (2016). 10.14814/phy2.12915 PMC5027349 27582065

[21] Kramer M (2013). Inhibition of H3K27 histone trimethylation activates fibroblasts and induces fibrosis. Ann Rheum Dis.

[22] Zhou, X. *et al*. Enhancer of Zeste Homolog 2 Inhibition Attenuates Renal Fibrosis by Maintaining Smad7 and Phosphatase and Tensin Homolog Expression. *J Am Soc Nephrol*, 10.1681/ASN.2015040457 (2015). 10.1681/ASN.2015040457 PMC4926973 26701983

[23] Fernandez-Catalan C (1998). Crystal structure of the complex formed by the membrane type 1-matrix metalloproteinase with the tissue inhibitor of metalloproteinases-2, the soluble progelatinase A receptor. EMBO J.

[24] Poincloux R, Lizarraga F, Chavrier P (2009). Matrix invasion by tumour cells: a focus on MT1-MMP trafficking to invadopodia. J Cell Sci.

[25] Decaneto E (2015). Pressure and Temperature Effects on the Activity and Structure of the Catalytic Domain of Human MT1-MMP. Biophys J.

[26] Worley JR (2003). Sequence motifs of tissue inhibitor of metalloproteinases 2 (TIMP-2) determining progelatinase A (proMMP-2) binding and activation by membrane-type metalloproteinase 1 (MT1-MMP). Biochem J.

[27] Chen WT, Wang JY (1999). Specialized surface protrusions of invasive cells, invadopodia and lamellipodia, have differential MT1-MMP, MMP-2, and TIMP-2 localization. Ann N Y Acad Sci.

[28] Zucker S (1998). Tissue inhibitor of metalloproteinase-2 (TIMP-2) binds to the catalytic domain of the cell surface receptor, membrane type 1-matrix metalloproteinase 1 (MT1-MMP). J Biol Chem.

[29] Bernardo MM, Fridman R (2003). TIMP-2 (tissue inhibitor of metalloproteinase-2) regulates MMP-2 (matrix metalloproteinase-2) activity in the extracellular environment after pro-MMP-2 activation by MT1 (membrane type 1)-MMP. Biochem J.

[30] Miranda TB (2009). DZNep is a global histone methylation inhibitor that reactivates developmental genes not silenced by DNA methylation. Mol Cancer Ther.

[31] Freese NH, Norris DC, Loraine AE (2016). Integrated genome browser: visual analytics platform for genomics. Bioinformatics.

[32] Kushida N (2016). Hypoxia-Inducible Factor-1alpha Activates the Transforming Growth Factor-beta/SMAD3 Pathway in Kidney Tubular Epithelial Cells. Am J Nephrol.

[33] Mimura I (2012). Dynamic Change of Chromatin Conformation in Response to Hypoxia Enhances the Expression of GLUT3 (SLC2A3) by Cooperative Interaction of Hypoxia-Inducible Factor 1 and KDM3A. Mol Cell Biol.

