# Supplementary material for: Genome-wide analysis revealed that DZNep reduces tubulointerstitial fibrosis via down-regulation of pro-fibrotic genes
Source: Sci Rep. 2018 Feb 28;8:3779. doi: 10.1038/s41598-018-22180-5 (PMC5830881; doi:10.1038/s41598-018-22180-5)
Supplement: Supplementary file 1 — Supplementary Information [file 41598_2018_22180_MOESM1_ESM.pdf]

Genome-wide analysis revealed that DZNep reduces tubulointerstitial fibrosis via down-regulation of pro-fibrotic genes.

Imari Mimura[1)], Yosuke Hirakawa[1)], Yasuharu Kanki[2)], Ryo Nakaki[3)], Yutaka Suzuki[4)], Tetsuhiro Tanaka[1)], Hiroyuki Aburatani[3)], Masaomi Nangaku[1)].

1) Division of Nephrology and Endocrinology, The University of Tokyo Graduate School of Medicine.

2) Isotope Science Center, The University of Tokyo.

3) Division of Genome Science, Research Center for Advanced Science and Technology, The University of Tokyo.

4) Graduate School of Frontier Sciences, The University of Tokyo.

*Corresponding authors: Masaomi Nangaku, M.D., Ph.D.,

Division of Nephrology and Endocrinology, The University of Tokyo Graduate School of Medicine, 7-3-1 Hongo, Bunkyo-ku, Tokyo, 113-8655.

Phone: +81-3-3815-5411 Fax: +81-3-5800-8860

E-mail: mnangaku-tky@umin.ac.jp

Running title: DZNep reduces renal fibrosis.

Key words: hypoxia; DZNep, tubulointerstitial fibrosis chronic kidney disease

Additional Information: No competing financial interests

Supplementary Legends.

Suppl. Table 1. List of genes in each group (Groups 1-3).

Suppl. Table 2. List of gene names shown in the Venn diagram (HK2 only, RPTEC only and commonly up-regulated under hypoxia)

Suppl. Table 3. List of primers used for qPCR.

Suppl. Table1.

Group1

Scfd1
Clic5
Gss
Rufy1
Zfp207
Guk1
Cd36
Chchd3
Ndufb10
1810020D17Rik
Hbb-b2
Hbb-b1
0610007C21Rik
Pth1r
Serpinf2
Mrpl16
Mtx1
Ttpa
Akr1b10
Nckap1
Enpp5
Hmbs
Gm10639
Pi4kb
Cyth2
Pigx
Acot1
Tm9sf1
Arpp19
Uba3
Folr1
Treh
Fam58b
Tpd52l1
Afg3l1
Ace
Crot
Copz2
3010026O09Rik
Igfbp3
Ccdc104
Tspan13
Hmgcr
Skiv2l2
5730469M10Rik
C1qtnf3
Gpihbp1
Hbb-b1
Maged1
Cd55
Hspa14
Fabp4
Slc16a4
Ipp
Necap2
Tmem30a
Gclc
Cyp7b1
Mpp6
Esm1
Id1
Nubpl
Nudt19
Scd1
Cyb561d2
Slco1a1
1810049H13Rik
Fkbp1a
Chst7
AU021092
Ufsp1
Prkaca
Isoc2b
Acp5
Gm14393
Pstk
Casc4
Mep1b
Csnk1g2
Als2cr4
Prkag1
Hba-a1
BC003331
Hp1bp3
Slco3a1
Aadat
Cd82
Lyrm1
Acsm3
Arsg
Fmo5
Spop
Wfdc15b
Agps
Cutc
Entpd5
Gm5918
Gm15137
Stk16
Mpv17l
3110045C21Rik
Ankrd24
Fam190a

Uqcrh	Srp9
Lyrm2	Fastk
Ubc	Dnase1
Chpt1	Pole4
Mrpl3	Gm10664
E130102H24Rik	Tomm40l
Pex11b	Hgd
Lamtor2	Txn2
Pcolce	Gcnt1
Osgepl1	Ccdc132
2210408F21Rik	
Afap1l1	
Cox7b	
Tdrd7	
Gyk	
Mff	
Hsd11b1	
Psmd13	
Egf	
Cd36	
Ppt2	
Dap3	
Abhd16a	
Rpl24	
Slc50a1	
2410022L05Rik	
Mcrs1	
1600012H06Rik	
Ccny	
RP23-3M10.11	
Afmid	
Ptpra	
Erbb2ip	
Hba-a2	
Pex3	
Slc12a1	

Group2

Bcl2l1
Ube2g2
C8g
Ppfibp1
Serpinb6a
Ccar1
Chac2
Dynll2
Mterfd1
Erbb2ip
Sap18
Hagh
Sec11c
Gm16286
Pdcd4
Fcgr2b
Slc12a1
Pcmtd2
2810403A07Rik
Sfpq
Abcf2
Fastk
Apoc3
Rnf44
Gmds
Upf3a
Hspb11
Mbl1
Jmjd6
Nt5dc1
Mrpl43
Synj2bp
Pkig
Cbs
Set
Myo1c
Dcaf11
Dohh
Serpina1c
D4Wsu53e
Akr1c19
Pop5
Ttc33
Fdps
Cyp4a32
Setd3
Serpina1a
Tmem116
Gpr112
Cldn10
D4Ertd22e
Trappc1
Dcn
Gamt
Tardbp
Sult1a1
0610037L13Rik
Folh1
Akap1
Chuk
Tmsb4x
Tsc22d3
Myo6
Pigp
Wbp1
Zfp467
Fmr1
Ftsj1
Phf10
Cd82
Serpina1f
5730469M10Rik
Gimap4
Qars
Arpc4
Usp8
Fbxo36
Eef1d
Hnf1a
1190007F08Rik
Ncoa6
Vim
2700029M09Rik
Uqcc
2210408F21Rik
Naa30
Fam36a
1810020D17Rik
Il11ra1
Prpf3
Miox
Osgep
Rangap1
0610007C21Rik
Pth1r
Usf1
Secisbp2l
Psmd11
Ssrp1
Hpn
5930416I19Rik
Abhd14a
Apoe

Group3

Lpar6
Jun
Rhob
Slc22a12
Thbd
Comt
Cd52
S100a6
Tcirg1
Tpm4
Cnn2
Hmox1
Timp1
Crbn
Bola1
Timp2
Rcn3
Slc9a3r2
2010111I01Rik
Cxcl14
Cldn1
Gcfc1
Serping1
Twf2
Pkdcc
Aldh1a7
Ldb1
Podxl
Casp4
Serpine2
Slamf9
Nphs2
Vim
Vcam1
Ptbp2
Ccnl2
Mxra8
Sparcl1
Anxa3
Abp1
Mgp
Coro1a
Ucp2
Nupr1
Bgn
Renbp
Eif4ebp1
Plat
Mak16
Herpud1
Tagln
Anxa2
Ccl5
Hspb8
Cdkn1c
Leng8
Zc3h7a
Slfn2
Lum
Wtip
Cxcl10
Cd53
Emp3
H2-Aa
H2-Ab1
Prkrip1
H2-DMa
Wdr45
Serpinb6a
Stap2
Stx16
Cald1
Fermt2
C1qb
Ifi47
Rgl2
Gucy1a3
Cd74
Lcn2
ORF61
Rbpms
Tsc22d3
Fxc1
Mdfic
Capg
Alox5ap
Rpl10
H2-Eb1
Cdh11
Il34
Sdccag3
Fcer1g
Ptprc
Col18a1
Icam1
Emr1
Igj
Csda
Lyz1
Marcks
Pcgf1
Sfrs18
Bcl2a1d
Anxa1
AW112010
Aimp2

Set	Ifi203	Vmp1
Evi2a	Arap1	Tardbp
Ighm	Rpl12	Wbscr27
Tmpo	Echdc2	Hmgcl
Lsp1	Acin1	Nmd3
Leprot	Gng10	Rbm17
Gm2023	Ucp2	Wsb1
Sparc	Tmem208	Hdac7
Clk4	Coq6	Ubxn6
Fbxo8	Slc5a12	Stk11
Sap25	Preb	Hnrpdl
Mndal	Eif4a1	Use1
Psme2	Llgl2	Uckl1
Arpc3	Cpxm1	Pex16
Pdcd6ip	Kif12	Stk16
D19Wsu162e	Slc50a1	Etl4
Cfh	Hyi	Flna
Tmsb4x	Aspdh	AI480526
Acsl4	Hnrpdl	Miox
Rab8b	Ech1	Stab1
Mrps18b	Lhfp	Bud31
Tmbim1	Arpc1b	Unc93b1
Tmsb10	Efemp1	Dcn
Taz	Gnb2l1	Axl
Mylk	Use1	Traf7
Nr3c1	Rbbp4	Cuedc2
Ranbp1	Rpl10a	Ccr2
Fam108c	Arpc5l	Ubxn8
Cdk9	Flna	Parp8
Mtcp1	Trim47	Commd6
Igfbp2	Npm1	Tubgcp3
Gm10639	Mpv17l	Spns2
Cpped1	Zfp40	Papola
Glis2	Ddx26b	Chkb
Plekho1	Adrm1	Morf4l2
Parp3	Nop56	Rnf213

Col3a1

Emilin1

Egfl7

Cp

H2-Q7

Neu1

Flot1

H2-Ke6

Egfl7

Psme1

H2-Q6

H2-Aa

Suppl. Table2.

HK2

AC009299.5
AC016739.2
ACAT2
ACOT7
ACSS2
ACTR3
AGRN
AHNAK
AHNAK2
AIF1L
AKR1B1
AMOTL2
ANKLE2
ANKRD10
ANP32B
ANXA3
ARF4
ARHGEF40
ARL6IP1
ARPC5
ASF1B
ATIC
ATP11A
ATP1B1
ATP2A2
ATXN2L
BIRC7
BNIP3
BNIP3P1
C11orf31
C12orf75
C7orf50
CACYBPP2
CALM1
CANX
CAPN2
CCND3
CCT2
CCT6A
CDC42EP3
CDK16
CDK2AP2
CDV3
CELF1
CHD3
CHMP3
CHRDL1
CLDN1
CLDN4
CLU
CNIH4
COL4A1
COL4A2
COL7A1
COL8A1
CRIM1
CRTAP
CTA-276O3.4
CTD-2116N17.1
CTPS1
CYB5B
DDX5
DEGS1
DERA
DERL2
DHCR24
DNM1
DNMT1
DUSP1
DYNLT1
EBP
EDC4
EIF4G2
ELF3
ENO2
EPCAM
ERGIC1
ESYT1
FADS1
FAM213A
FAM57A
FAM65A
FANCA
FARP1
FASN
FAXC
FBXO2
FDFT1
FDPS
FGB
FKBP9
FLII
FLNC
FLOT1
FUS
FXYD2
GABARAPL1
GALE
GATAD2A
GLS
GNB1
GOT1
GPC4
GPRC5A
GUCD1
H2AFY

H3F3AP4
HAVCR1
HBEGF
HDAC2
HIATL1
HK1
HMGA1
HMGB1P5
HMGB2
HMGN1P38
HMGN2
HMGN2P5
HNF1B
HNRNPA1P48
HNRNPA2B1
HNRNPAB
HNRNPD
HNRNPDL
HNRNPH1
HNRNPM
HNRNPU
HNRNPUP1
HP1BP3
HSP90AA1
HSP90B1
HSPB8
HSPD1
HSPG2
HTATIP2
HYI
IDH2
IGF2R
IL11
IMPA2
IMPDH2
INSIG1
ITGB1BP1
ITGB3
JUND
KDELC1
KIAA1191
KIFC1
KLC1
KPNA2
KPNB1
KRT80
LAMA5
LAMC1
LDHA
LDHAP4
LDLR
LEMD2
LEPROT
LGALS1
LHX1
LIF
LIMK1
LINC00958
LITAF
LMCD1
LMNB2
LOXL1
LPCAT1
LRRC61
LRRC8A
LRRN4
LSM14B
LSR
LSS
LTBP4
MAD1L1
MAPRE1
MARVELD1
MAT2A
MCM3
Metazoa_SRP
MFI2
MGLL
MGMT
MICAL2
MICB
MMAB
MMP14
MMP24
MOV10
MPC2
MPZL1
MSANTD3
MSMO1
MT1F
MTCH2
MTHFD1
MTND1P23
MT-ND5
MT-ND6
MVD
MXRA7
MYBL2
MYO19
MYO1E
MYOF
NAP1L1
NAP1L4
NBL1
NEK6
NES
NFE2L1
NFKBIA

NNMT
NPM1
NRM
NUAK2
NUDT21
NXN
OCIAD1
OCIAD2
P3H2
PABPC1
PABPC4
PALLD
PAM
PAX8
PCNA
PDZK1IP1
PFKP
PFN2
PGLS
PGM1
PGRMC1
PHGDH
PLEC
PLEKHA2
PLPP2
POLE
POLR2A
PPFIBP1
PPP1CB
PPP1CC
PRDX5
PRKACA
PRKAR1A
PRKCZ
PRRC2B
PSMA1
PSMA3
PSMD11
PTGES3
PTGES3P1
PTMS
PTPRF
PXMP2
RBBP4
RBCK1
RBMX
RBP7
RHOD
RHOF
RN7SL1
RNF4
RP11-252P18.1
RP11-312J18.5
RP11-333E13.2
RP11-69L16.5
RP11-982M15.2
RP1-241P17.4
RPA1
RPL12
RPL12P4
RPS12
RPS21P4
RRAS
RRM2
RTN4
S100A2
SCD
SCDP1
SEPT8
SEPT9
SETP14
SFPQ
SHMT1
SLC38A2
SLCO4A1
SNHG6
SPCS3
SPTAN1
SPTBN1
SQLE
SREBF2
SRSF1
SRSF3
SRSF5
SRSF6
SRSF7
SSC4D
STAG3
STAT1
STAT2
TAPBP
TARDBP
TBCD
TCEA3
TFPI2
TGIF1
THOP1
TIMP2
TMEM139
TMEM14DP
TMEM184B
TMEM256
TMEM54
TMEM97
TMEM98
TMPO
TNFRSF12A
TPM4

TPT1
TPT1P9
TRA2B
TRAM1
TRIB3
TRNP1
TXNRD1
UBAC2
UBE2A
UBE2D2
UBE2D3
UBE2MP1
UBE2SP1
UBE2SP2
UFD1L
UGCG
UGDH
UXS1
VCP
WHSC1
WSB2
WWC1
WWTR1
XRCC5
Y_RNA
YWHAB
YWHAH
YWHAZP3
ZDHHC7
ZFAND3
ZFYVE21
ZWINT

RPTEC

A4GALT
AAAS
AACS
AAMP
AARS
ABHD12
ABHD14B
ABL1
ABR
AC004057.1
AC004453.8
AC005943.2
AC007969.5
AC009133.22
AC016734.2
AC016739.2
AC079250.1
AC108479.2
AC111200.7
ACADVL
ACAT2
ACLY
ACOT7
ACSS2
ACTR1A
ACY1
ADAM15
ADCK4
ADD1
ADGRG1
ADIPOR1
ADRM1
ADSL
AEBP1
AES
AGAP3
AGPAT1
AGPAT3
AGRN
AGTRAP
AHCY
AHNAK
AIF1L
AIP
AK1
AK2
AKR1A1
AKR1B1
AKR7A2
AKT1
AKT1S1
AKT2
ALAS1
ALDOA
ALG3
ALKBH7
ALYREF
AMFR
AMPD2
ANAPC11
ANAPC15
ANGPTL4
ANKRD52
ANPEP
ANXA11
AP1B1
AP1M1
AP1M2
AP2A2
AP2S1
AP3D1
APEH
APH1A
APOA1BP
APOBEC3C
APOC1
APOE
APRT
ARF3
ARF5
ARFGAP1
ARFIP2
ARHGAP22
ARHGEF1
ARL16
ARL2
ARL4C
ARL6IP4
ARMC6
ARPC1A
ARPC1B
ARPC4
ARRDC1
ASF1B
ASNA1
ASS1
ATG4B
ATIC
ATOX1
ATP2A2
ATP5B
ATP5G1
ATP5G2
ATP5G2P4
ATP5G3
ATP5I

ATP5J2	BTBD2	CARM1
ATP5L	BTBD6	CARS2
ATP5LP2	C11orf31	CAST
ATP6V0B	C11orf84	CBX6
ATP6V0C	C11orf98	CCAR2
ATP6V0D1	C12orf10	CCDC124
ATP6V0E2	C12orf57	CCDC167
ATP6V1F	C12orf75	CCM2
ATXN2L	C14orf1	CCND3
ATXN7L3	C14orf159	CD151
AUP1	C16orf13	CD276
AURKAIP1	C17orf62	CD2BP2
AURKB	C17orf89	CD40
B4GALT2	C19orf24	CD44
B4GALT7	C19orf33	CD63
B9D1	C19orf48	CD70
BABAM1	C19orf60	CD99
BACE2	C1orf122	CDC20
BAD	C1orf43	CDC34
BAG6	C1QBP	CDC42EP1
BANF1	C20orf24	CDCA3
BAX	C21orf33	CDCP1
BCAP31	C3	CDH6
BCL2L1	C6orf1	CDIP1
BCL7B	C7orf49	CDK16
BCL7C	C7orf50	CDK2AP2
BID	C9orf69	CDK4
BIRC5	CALM3	CDK9
BLOC1S1	CALR	CDKN1A
BLVRB	CANX	CDKN2A
BOK	CAPG	CENPM
BOLA2B	CAPN1	CERCAM
BOP1	CAPN2	CERS2
BRMS1	CAPNS1	CFL1
BSG	CARD19	CH507-513H4.6
BST2	CARHSP1	CH507-9B2.3

CHCHD10
CHCHD2
CHCHD5
CHKB
CHMP1A
CHMP3
CHPF
CHURC1
CIAO1
CIB1
CISD3
CKAP4
CKLF
CKS2
CLCN7
CLDN4
CLIC1
CLK3
CLPTM1L
CLTA
CLTB
CLU
CMC2
CMTM3
CNIH4
CNN2
CNP
CNPY2
CNPY3
COA3
COASY
COL18A1
COMMD4
COMMD5
COMTD1
COPE
COPS6
COPZ1
COQ9
CORO1B
COTL1
COX14
COX17
COX4I1
COX5A
COX6B1
COX7A2
COX7A2L
COX7B
COX8A
CPNE2
CPSF3L
CRAT
CREB3
CRIP1
CRIP2
CRLS1
CRTAP
CRYAB
CS
CSK
CSNK1G2
CSNK2B
CTA-276O3.4
CTB-33G10.1
CTB-63M22.1
CTBP1
CTC-260E6.2
CTC-575D19.1
CTD-2192J16.15
CTD-2270N23.1
CTHRC1
CUEDC1
CYB561A3
CYBA
CYC1
CYP27A1
CYS1
CYTH2
DALRD3
DANCR
DAP
DAPK3
DAZAP1
DBI
DBNDD1
DBNDD2
DCTN2
DCTN3
DCTPP1
DDA1
DDAH2
DDB1
DDOST
DDX39A
DDX54
DEAF1
DESI1
DGCR6L
DGUOK
DHCR24
DHCR7
DHPS
DHX30
DMKN
DMTN
DNAAF5
DNAJA3

DNAJB11	EIF3G	EVA1B
DNAJC5	EIF3K	EXOSC4
DNM2	EIF4G2	FAAP20
DNPEP	EIF5A	FADD
DNPH1	EIF5AL1	FADS1
DNTTIP1	EIF5AP4	FADS2
DOC2B	EIF6	FAHD2A
DPAGT1	ELAVL1	FAM101B
DPF2	ELF3	FAM134A
DPM2	ELOF1	FAM213B
DPP3	ELOVL1	FAM214B
DRAP1	ELP6	FAM3A
DTYMK	EMC10	FAM50A
DUS1L	EMC6	FAM57A
DUT	EMD	FAM65A
DVL1	EML3	FAM96B
E2F4	EMP3	FARP1
EBP	ENO1	FARSA
ECE1	ENO1P1	FASN
ECH1	ENSA	FASTK
ECHS1	ENSAP2	FAT1
ECI1	ENTPD6	FAU
EDF1	ENY2	FAUP1
EEF1D	EPHA2	FBL
EEF1G	EPHB2	FBLIM1
EEF2	EPHB4	FBN1
EFEMP2	EPN1	FBRSL1
EFHD2	ERBB2	FDFT1
EFTUD2	ERCC1	FDPS
EHD1	ERCC2	FDX1L
EHD4	ERF	FGFR1
EIF1	ERI3	FGFRL1
EIF3B	ESYT1	FIBP
EIF3C	ETFB	FIS1
EIF3CL	ETHE1	FKBP2
EIF3FP3	EVA1A	FKBP4

FKBP8	GCHFR	GUCD1
FKBP9	GCSHP5	GUK1
FLAD1	GDI1	H2AFJ
FLII	GET4	H2AFV
FLOT1	GFER	H2AFX
FLOT2	GGA1	H2AFY
FLYWCH1	GGT1	HADHA
FLYWCH2	GIPC1	HAGH
FOSL1	GLB1	HAX1
FPGS	GLTSCR2	HDGF
FSCN1	GMPPB	HGS
FSTL3	GNAI2	HINT1
FUOM	GNAS	HINT2
FURIN	GNB1	HIP1R
FUS	GNB2	HM13
FXYD2	GNG11	HMBS
FXYD5	GNG5	HMG20B
FXYD6	GPAA1	HMGA1
FZR1	GPI	HMGA1P2
G6PC3	GPR108	HMGA1P8
GABARAP	GPS1	HMGN1P38
GADD45GIP1	GPS2	HMGN2
GAK	GPT2	HMOX2
GAL3ST1	GPX1	HN1
GALE	GPX1P1	HNF1B
GALK1	GPX4	HNRNPAB
GALNT14	GRAMD1A	HNRNPD
GALNT2	GRHPR	HNRNPH1
GAMT	GRWD1	HNRNPU
GANAB	GSK3A	HNRNPUL1
GAPDH	GSPT1	HOMER3
GAPDHP1	GSS	HOXB2
GAPDHP65	GSTP1	HPCAL1
GAPDHP72	GTF2F1	HRAS
GAS6	GTF3C5	HSD17B10
GATAD2A	GTPBP2	HSD3B7

HSP90AB1
HSP90B1
HSPA5
HSPB8
HSPBP1
HSPD1
HSPE1
HSPE1P2
HSPG2
HYAL2
HYI
HYOU1
ICAM1
IDH2
IDH3G
IER2
IFI27L2
IFITM1
IFITM2
IFRD2
IGFBP2
IGFBP6
IGFBP7
IGSF8
IL32
ILK
ILVBL
IMPA2
IMPDH1
IMPDH2
INF2
INO80E
IPO4
IRAK1
IRF3
IRF9
ISOC2
ISYNA1
ITGA3
ITGB1BP1
ITGB5
ITPA
JOSD2
JPX_1
JTB
JUND
JUP
KCNJ15
KCNK3
KCTD11
KDELR1
KEAP1
KIAA0141
KIAA2013
KIF22
KLHDC3
KPNB1
KRT18
KRT19
KRTCAP2
LAMB1
LAMC1
LAMTOR1
LASP1
LDLR
LEMD2
LGALS1
LIMD2
LIMK1
LINC01578
LITAF
LMF2
LMNA
LMNB2
LONP1
LOXL1
LOXL2
LPCAT4
LRP10
LRPAP1
LRRC61
LRRC75A-AS1
LRRN4
LSM10
LSM2
LSM4
LSM5
LSM7
LSR
LSS
LTBP2
LTBP3
LTBP4
LYPLA2
LZTR1
MACF1
MAEA
MAF1
MAGED1
MAL
MALSU1
MAN2B1
MAN2C1
MANF
MAP2K1
MAP2K2
MAP3K11
MAP7D1

MAPK13	MMP14	MXD4
MAPKAP1	MOB3A	MYADM
MAPKAPK2	MOSPD3	MYBL2
MAPKAPK3	MPC2	MYDGF
MARCH2	MPDU1	MYL6
MARS	MPG	MYL6B
MARVELD1	MPRIP	MYO1C
MAZ	MPST	MYOF
MBD3	MRPL11	MZT2A
MBOAT7	MRPL12	MZT2B
MCM4	MRPL14	NAA10
MCM5	MRPL17	NACC1
MCM7	MRPL2	NADSYN1
MCRS1	MRPL21	NAGLU
MCTS1	MRPL23	NANS
MDH2	MRPL28	NAP1L1
MDK	MRPL4	NAPA
MED25	MRPL52	NARF
METRN	MRPS11	NBL1
MEX3D	MRPS16	NCLN
MFAP2	MRPS18A	NCS1
MFSD10	MRPS6	NCSTN
MFSD12	MRTO4	NDUFA1
MGAT4B	MT1F	NDUFA10
MGLL	MT1G	NDUFA11
MGMT	MTA1	NDUFA13
MGST3	MTA2	NDUFA3
MICALL1	MT-ATP6	NDUFA7
MIF	MTATP6P1	NDUFA8
MINOS1	MTCH1	NDUFB1
MIR210HG	MTCH2	NDUFB10
MKNK2	MT-CO2	NDUFB11
MLEC	MTG1	NDUFB2
MLF2	MVD	NDUFB3
MLPH	MVK	NDUFB7
MMAB	MVP	NDUFB8

NDUFB9	NT5E	PAX8
NDUFC2	NTMT1	PCBD1
NDUFS5	NUBP2	PCBP2
NDUFS6	NUDC	PCBP4
NDUFS8	NUDCD3	PCCB
NDUFV1	NUDT1	PCDHGC3
NEDD8	NUDT14	PCK2
NEFL	NUDT16L1	PCYT2
NEK6	NUDT22	PDAP1
NELFB	NUTF2	PDCD2
NELFCD	NXN	PDGFB
NENF	OAZ1	PDIA4
NEU1	OAZ2	PDLIM4
NF2	OBSL1	PDLIM7
NFE2L1	OCIAD2	PDXK
NHP2	OGDH	PDZK1IP1
NHP2P1	ORAI2	PEF1
NIPSNAP1	ORAI3	PEMT
NKIRAS2	ORMDL3	PEPD
NME1	OXLD1	PEX10
NME2	P3H3	PFDN2
NME2P1	P3H4	PFDN5
NME4	P4HB	PFDN6
NMT1	PA2G4	PFKP
NNMT	PA2G4P4	PFN1
NOC2L	PABPC1	PFN1P1
NOMO2	PABPC4	PFN2
NOSIP	PABPN1	PGAM5
NPDC1	PACS1	PGD
NPLOC4	PACS2	PGLS
NPM3	PADI2	PGM1
NR2F6	PAFAH1B3	PHB
NRBP1	PAM	PHB2
NRM	PARP10	PHC2
NRSN2	PARVA	PHF19
NT5DC2	PARVB	PHF23

PHF5A	POLD4	PRR13
PHGDH	POLDIP3	PRRC2A
PHLDA2	POLE4	PRRC2B
PHPT1	POLR1D	PSENEN
PIEZO1	POLR2A	PSMA7
PIGQ	POLR2E	PSMB10
PIGT	POLR2J	PSMB3
PIH1D1	POMGNT1	PSMB4
PIK3R2	POMP	PSMB6
PIN1	POP7	PSMC3
PITPNA	PPDPF	PSMC4
PITPNM1	PPIB	PSMD13
PKM	PPP1CA	PSMD2
PKN1	PPP1R11	PSMD3
PLA2G16	PPP1R13L	PSMD8
PLAT	PPP1R14B	PSME1
PLAU	PPP1R14BP3	PSME3
PLAUR	PPP2R1A	PSMG3
PLCD3	PPP2R4	PSPH
PLEC	PPP2R5D	PTBP1
PLEKHG3	PPP4C	PTGES2
PLEKHJ1	PPP5C	PTGES3
PLGRKT	PPP6R1	PTGES3P1
PLK1	PQBP1	PTMS
PLOD3	PQLC1	PTOV1
PLP2	PRDX2	PTPMT1
PLPP2	PRDX5	PTPRF
PLPP4	PREB	PTPRU
PLTP	PRELID1	PUF60
PLXNB1	PRKACA	PVR
PMM1	PRKCDBP	PVRL2
PMPCA	PRKCSH	PVT1
PMVK	PRMT1	PXN
PNKP	PRMT2	PYCARD
PNP	PROSER2	PYCR2
PNPLA6	PRPF19	QARS

QPRT
R3HDM4
RAB1B
RAB34
RAB35
RAB5C
RAB8A
RABAC1
RABL6
RAD23A
RALY
RANBP3
RANGAP1
RARA
RASSF7
RBCK1
RBM14
RBM4
RBM42
RCC2
REC8
REEP5
RELA
RER1
RERE
RGL2
RGS3
RHBDF2
RHOA
RHOC
RHOF
RNASEH2A
RNASEK
RNF10
RNF121
RNF126
RNF167
RNF181
RNF187
RNF220
RNF26
RNF5
RNF5P1
RNH1
RNPEP
RNPEPL1
RNPS1
RNPS1P1
ROMO1
RP11-112J1.1
RP11-12M9.3
RP11-170M17.2
RP11-175B9.3
RP11-252P18.1
RP11-270C12.3
RP11-289I10.2
RP11-290L1.3
RP11-333E13.2
RP11-343C2.11
RP11-365O16.1
RP11-36C20.1
RP11-371A22.1
RP11-400N13.3
RP11-425L10.1
RP11-466H18.1
RP11-475C16.1
RP11-478C6.4
RP11-492M23.2
RP11-51O6.1
RP11-543P15.1
RP11-572P18.1
RP1-159A19.3
RP11-69L16.5
RP11-761N21.2
RP11-778D9.4
RP11-796G6.1
RP11-864N7.2
RP11-982M15.2
RP1-278E11.3
RP13-608F4.5
RP3-340B19.2
RP3-486I3.4
RP5-827C21.1
RPL10
RPL12P12
RPL12P16
RPL12P4
RPL13
RPL13A
RPL13AP25
RPL13AP5
RPL13AP6
RPL13AP7
RPL13P12
RPL15
RPL15P3
RPL17
RPL18
RPL18A
RPL18AP3
RPL21P119
RPL21P75
RPL22L1
RPL23
RPL24P4
RPL26
RPL26P19
RPL27

RPL27A	RPS11	RPSAP12
RPL28	RPS13	RPSAP15
RPL3	RPS14	RPSAP18
RPL30	RPS15	RPSAP19
RPL31	RPS15A	RPSAP54
RPL31P63	RPS15AP1	RPSAP58
RPL32	RPS15AP24	RRAS
RPL34	RPS16	RRP7A
RPL34P18	RPS17	RTKN
RPL35	RPS18P12	RUSC1
RPL35A	RPS18P9	RUVBL2
RPL35P2	RPS19	S100A10
RPL35P5	RPS2	S100A11
RPL37	RPS20	S100A13
RPL37A	RPS20P14	S100A16
RPL37P2	RPS21	S100A2
RPL37P23	RPS21P4	S100A6
RPL38	RPS23	SAE1
RPL39	RPS23P8	SAMD1
RPL39P3	RPS27	SAMD4B
RPL3P4	RPS27A	SAP30BP
RPL7A	RPS28P7	SAT2
RPL7AP30	RPS29	SBF1
RPL7AP6	RPS2P46	SC22CB-1E7.1
RPL7AP66	RPS2P5	SCAF1
RPL8	RPS2P55	SCAMP2
RPL9P7	RPS3	SCAMP3
RPL9P9	RPS3AP47	SCAMP4
RPLP0	RPS3AP6	SCARB1
RPLP0P6	RPS5	SCO2
RPLP1	RPS6KA4	SCRIB
RPLP2	RPS7	SCYL1
RPP21	RPS7P1	SDC1
RPS10	RPS7P11	SDHA
RPS10-NUDT3	RPS9	SEC61G
RPS10P3	RPSA	SELK

SEMA7A
SENP3
SEPN1
SEPT2P1
SEPT9
SEPW1
SERF2
SERP1
SERPINA1
SERPINB6
SERPINH1
SESN2
SF1
SF3A1
SF3A2
SF3B2
SF3B3
SF3B4
SF3B5
SFN
SFXN3
SGTA
SH3BP2
SH3GL1
SH3GLB2
SHARPIN
SHISA5
SHKBP1
SHMT1
SHMT2
SIGIRR
SIGLEC15
SIGMAR1
SIL1
SIPA1
SIRPA
SIRPB1
SIVA1
SLC10A3
SLC12A4
SLC16A3
SLC1A5
SLC25A1
SLC25A10
SLC25A11
SLC25A23
SLC25A39
SLC25A6
SLC25A6
SLC29A1
SLC2A4RG
SLC35A2
SLC35B2
SLC35C2
SLC35F6
SLC37A4
SLC39A1
SLC39A13
SLC39A4
SLC44A2
SLC4A2
SLC50A1
SLC52A2
SLC6A6
SLC7A5
SLCO4A1
SLIRP
SMAGP
SMARCB1
SMARCD1
SMARCD2
SMDT1
SMIM12
SMOX
SMTN
SMUG1
SND1
SNHG5
SNHG6
SNHG8
SNRNP25
SNRNP70
SNRPA
SNRPB
SNRPD2
SNRPEP4
SNRPF
SNRPFP1
SNRPG
SNRPGP10
SNRPGP15
SNRPN
SNX17
SNX8
SORBS3
SPAG7
SPINT1
SPINT2
SPNS2
SPTAN1
SPTBN1
SQRDL
SQSTM1
SREBF1
SREBF2
SRM
SRPR
SSH3

SSNA1	TARBP2	TMEM129
SSR4	TBCB	TMEM134
SSU72	TBCD	TMEM141
STAP2	TBL3	TMEM147
STARD3	TBRG4	TMEM14C
STEAP3	TCEB2	TMEM160
STIM1	TCF25	TMEM18
STIP1	TCF3	TMEM184B
STK11	TCIRG1	TMEM189
STMN3	TCP1	TMEM205
STOML2	TECR	TMEM256
STRA13	TECRP1	TMEM256-PLSCR3
STRN4	TFPI2	TMEM259
STUB1	TGFB1	TMEM51
STX10	TGIF1	TMEM54
STXBP2	TGM2	TMEM63B
STYXL1	THAP4	TMEM8A
SUB1	THOC6	TMEM9
SUMF2	THOP1	TMSB4XP8
SUMO3	TIMM10	TMUB1
SUN1	TIMM17B	TNFAIP2
SUN2	TIMM50	TNFRSF10B
SURF1	TIMP2	TNFRSF12A
SURF4	TINAGL1	TNFRSF6B
SYDE1	TK1	TNIP1
SYMPK	TKFC	TNKS1BP1
SYNGR2	TKT	TOLLIP
TACC3	TLDC1	TOMM40
TADA3	TM4SF1	TOMM5
TAF10	TM7SF2	TOMM7
TAF15	TMED1	TP53
TAF6	TMED3	TP53I13
TAGLN	TMEM101	TPD52L2
TAGLN2	TMEM11	TPGS2
TAGLN2P1	TMEM120A	TPM2
TAPBP	TMEM127	TRABD

TRAF4	TUBGCP2	USP22
TRAF7	TUFM	USP5
TRAP1	TUSC2	VAMP5
TRAPPC5	TWF2	VARS
TRIB3	TXN	VASP
TRIM28	TXNL4A	VAT1
TRIM8	TYMP	VCAN
TRIOBP	TYMS	VCP
TRMT112	U2AF1	VEGFB
TRMT2A	U2AF1L5	VPS25
TRNP1	U2AF2	VPS51
TRPT1	UBA1	VPS72
TSFM	UBA52	WARS
TSKU	UBAC2	WASF2
TSPAN1	UBE2A	WBP1
TSPAN14	UBE2I	WBP2
TSPAN17	UBE2M	WDR18
TSPAN4	UBE2MP1	WDR34
TSPAN9	UBE2Q1	WDR45
TSPO	UBE2SP1	WDR54
TSR3	UBE2SP2	WDR74
TST	UBL5	WDR77
TSTA3	UBTD1	WIPI2
TSTD1	UBXN1	WWTR1
TTLL12	UCHL1	XBP1
TTYH3	UFD1L	YBX3
TUBA1A	UPP1	YIF1A
TUBA1B	UQCC2	YIF1B
TUBA1C	UQCR11	YKT6
TUBA4A	UQCRB	YWHAB
TUBB	UQCRC1	ZDHHC12
TUBB2B	UQCRQ	ZDHHC16
TUBB3	UROD	ZDHHC3
TUBB4B	USF1	ZDHHC5
TUBB6	USF2	ZDHHC7
TUBBP1	USMG5	ZDHHC8

ZFAND3

ZFAS1

ZFPL1

ZFYVE19

ZFYVE21

ZMAT5

ZNF358

ZNF511

ZNF581

ZNHIT1

ZSWIM8

ZYX

Common

AC016739.2
ACAT2
ACOT7
ACSS2
AGRN
AHNAK
AIF1L
AKR1B1
ASF1B
ATIC
ATP2A2
ATXN2L
C11orf31
C12orf75
C7orf50
CANX
CAPN2
CCND3
CDK16
CDK2AP2
CHMP3
CLDN4
CLU
CNIH4
CRTAP
CTA-276O3.4
DHCR24
EBP
EIF4G2
ELF3
ESYT1
FADS1
FAM57A
FAM65A
FARP1
FASN
FDFT1
FDPS
FKBP9
FLII
FLOT1
FUS
FXYD2
GALE
GATAD2A
GNB1
GUCD1
H2AFY
HMGA1
HMGN1P38
HMGN2
HNF1B
HNRNPAB
HNRNPD
HNRNPH1
HNRNPU
HSP90B1
HSPB8
HSPD1
HSPG2
HYI
IDH2
IMPA2
IMPDH2
ITGB1BP1
JUND
KPNB1
LAMC1
LDLR
LEMD2
LGALS1
LIMK1
LITAF
LMNB2
LOXL1
LRRC61
LRRN4
LSR
LSS
LTBP4
MARVELD1
MGLL
MGMT
MMAB
MMP14
MPC2
MT1F
MTCH2
MVD
MYBL2
MYOF
NAP1L1
NBL1
NEK6
NFE2L1
NNMT
NRM
NXN
OCIAD2
PABPC1
PABPC4
PAM
PAX8
PDZK1IP1
PFKP
PFN2

PGLS
PGM1
PHGDH
PLEC
PLPP2
POLR2A
PRDX5
PRKACA
PRRC2B
PTGES3
PTGES3P1
PTMS
PTPRF
RBCK1
RHOF
RP11-252P18.1
RP11-333E13.2
RP11-69L16.5
RP11-982M15.2
RPL12P4
RPS21P4
RRAS
S100A2
SEPT9
SHMT1
SLCO4A1
SNHG6
SPTAN1
SPTBN1
SREBF2
TAPBP
TBCD
TFPI2
TGIF1
THOP1
TIMP2
TMEM184B
TMEM256
TMEM54
TNFRSF12A
TRIB3
TRNP1
UBAC2
UBE2A
UBE2MP1
UBE2SP1
UBE2SP2
UFD1L
VCP
WWTR1
YWHAB
ZDHHC7
ZFAND3
ZFYVE21

Supplementary Table 3.

gene name	Species		
Col3a1	mouse	Forward	AGCCTTCTACACCTGCTCCT
		Reverse	CGGATAGCCACCCATTCCTC
Timp2	mouse	Forward	GACTTCATTGTGCCCTGGGA
		Reverse	TGGTGCCCATTGATGCTCTT
βActin	mouse	Forward	TGAGGTACTAGCCACGAGAGAG
		Reverse	ACACCCGCCACCAGGTAAGCA
TIMP2	human	Forward	TCCTCTTGATAGGGTTGCCA
		Reverse	CGTTTTGCAATGCAGATGTA
βACTB	human	Forward	AACTGGTCTCAAGTCAGTGTACAGG
		Reverse	TCCCCCAACTTGAGATGTATGAAG
